# Effect of temperature change on synaptic transmission at crayfish neuromuscular junctions

**DOI:** 10.1242/bio.037820

**Published:** 2018-11-07

**Authors:** Yuechen Zhu, Leo de Castro, Robin Lewis Cooper

**Affiliations:** 1 Department of Biology, University of Kentucky, Lexington, KY 40506-0225, USA; 2 Massachusetts Institute of Technology, Electrical Engineering and Computer Science (EECS), 50 Vassar St, Cambridge, MA 02142, USA

**Keywords:** Invertebrates, Crustacean, Adaptation, Modulation, Synaptic transmission

## Abstract

Ectothermic animals in areas characterised by seasonal changes are susceptible to extreme fluctuations in temperature. To survive through varied temperatures, ectotherms have developed unique strategies. This study focuses on synaptic transmission function at cold temperatures, as it is a vital component of ectothermic animals' survival. For determining how synaptic transmission is influenced by an acute change in temperature (20°C to 10°C within a minute) and chronic cold (10°C), the crayfish (*Procambarus clarkii*) neuromuscular junction (NMJ) was used as a model. To simulate chronic cold conditions, crayfish were acclimated to 15°C for 1 week and then to 10°C for 1 week. They were then used to examine the synaptic properties associated with the low output nerve terminals on the opener muscle in the walking legs and high output innervation on the abdominal deep extensor muscle. The excitatory postsynaptic potentials (EPSPs) of the opener NMJs increased in amplitude with acute warming (20°C) after being acclimated to cold; however, the deep extensor muscles showed varied changes in EPSP amplitude. Synaptic transmission at both NMJs was enhanced with exposure to the modulators serotonin or octopamine. The membrane resistance of the muscles decreased 33% and the resting membrane potential hyperpolarised upon warm exposure. Analysis of haemolymph indicated that octopamine increases during cold exposure. These results suggest bioamine modulation as a possible mechanism for ensuring that synaptic transmission remains functional at low temperatures.

## Abstract (precis)

**Summary:** Studies were conducted in the model invertebrate, the crayfish, to determine the effects of the acute change from 20°C to 10°C and vice versa on synaptic transmission.

## INTRODUCTION

The effect of temperature on neuronal function has been of interest for years, as there are fundamental questions about how various ectothermic animals can adapt to extreme temperatures and, in some cases, live in conditions where other ectotherms cannot (Chung et al., 2012; Somero et al., 1996; Städele et al., 2015; Veselý et al., 2015). There is also an interest in understanding how cold affects neural tissue when inducing abnormal cold conditions in endotherms (humans) for therapeutic reasons (Jin et al., 2014; Mourvillier et al., 2013). Intact neural systems within whole organisms are too complex to address all the various factors induced by temperature changes since each cell may vary in terms of its membrane properties, ion channels, synaptic actions and biochemical cellular responses, which all may alter the excitability of neurons. However, using model neural circuits and synaptic preparations, such as the stomatogastric nervous system and neuromuscular junctions of crustaceans, fundamental questions of the biological effects can be addressed (Städele et al., 2015). This study focused on the effects of acute and moderate conditioning to cold on synaptic transmission at neuromuscular junctions (NMJs). We used a robust crustacean species (crayfish, *Procambarus clarkii*) that can live in nearly freezing conditions for short periods (i.e. minutes) and in warm seasonal conditions (40°C) in nature. The crayfish (*P. clarkii*) is a successful ectothermic species that can adjust to different environments. It is native to subtropical climates but can live across different temperature zones and is known as a robust invasive species throughout North America, Europe and Asia (Foster and Harper, 2007; Gherardi, 2010; Yue et al., 2010).

In addressing the fundamental synaptic alterations that occur with temperature change in defined synaptic NMJ preparations, a basic understanding can be gained that can help to aid in addressing how complex neural networks in intact nervous systems function with temperature changes (Katz, 1949; Katz and Kuffler, 1946; Städele et al., 2015). NMJs have served as models for years in addressing synaptic function; however, there are also limitations in using models to address neuron-to-neuron synaptic communication. The crayfish NMJ is unique as a synaptic model since the evoked responses are graded in synaptic strength and can show synaptic facilitation and/or depression. The motor neurons utilise glutamate or GABA as a neurotransmitter, which the postsynaptic muscle must integrate, much like a dendrite of a postsynaptic neuron (Atwood, 1982a,b; Atwood and Cooper, 1996; see review by Titlow and Cooper, 2018).

Two synaptic neuromuscular junctions were chosen to investigate the effects of rapid and acclimated cold temperature since they differ in synaptic efficacy. The innervation of the opener muscle (slow muscle phenotype) is tonic in nature with low-output synapses and the deep lateral extensor muscle (DEL1) is a fast phenotype muscle with phasic, high-output synaptic innervation. Previous studies of the properties of these two crustacean neuromuscular preparations have been conducted at room temperature (Atwood, 1976; Atwood and Cooper, 1996; Cooper and Cooper, 2009).

Modulators are important factors for inducing physiological changes to allow arthropods to respond to different environments. Modulators, such as serotonin (5-HT), octopamine (OA), dopamine (DA) and peptides are well known to alter neuronal, cardiac, GI, ventilatory and skeletal muscle function in crustaceans and insects (Listerman et al., 2000; Strawn et al., 2000; review by Cooper et al., 2011a,b; Shuranova et al., 2006; Städele et al., 2015). Octopamine produces long-lasting contracture in exoskeletal muscles in lobsters and can have direct effects on muscle (Evans et al., 1975). 5-HT also enhances synapse output and muscle tension generation in a temperature-dependent manner in lobsters (Hamilton et al., 2007). Peptides are known to influence NMJ function; for example, DF2 (DRNFLRFamide) enhances synapse efficacy in crustaceans (Friedrich et al., 1994) and tachykinin-related peptide Ia (CabTRP Ia) alters neuronal function to likely help compensate for neural circuit activity during cold exposure (Städele et al., 2015).

As previous studies have shown, 5-HT and OA enhance synaptic transmission in the opener and DEL1 muscles in the crayfish; however, these studies did not focus on the actions of the modulators in temperatures colder than 20°C (Djokaj et al., 2001) and did not examine crayfish acclimated to lower temperatures. Both modulators are known to have a wide range of effects in crustaceans (see review by Shuranova et al., 2006). Considering that 5-HT and OA rapidly alter synaptic transmission centrally and at NMJs, it would not be surprising for thermal stress to result in acute changes and have potentially long-term effects in influencing synaptic efficacy. 5-HT and OA have both been shown to alter social behaviours in crayfish (Huber and Delago, 1998; Listerman et al., 2000; Livingston et al., 1980; McRae, 1996) and are known to be rapidly altered in the haemolymph due to whole animal activity in crabs (Sneddon et al., 2000) and insects (Hirashima and Eto, 1993).

The purpose of this study was to understand how these two model NMJs function with acute cold exposure and relatively chronic cold conditioning. In addition, we wanted to address how these preparations would respond to modulation by two commonly assessed biogenic amines (5-HT and OA) from cold-acclimated crayfish. We postulated that 5-HT and OA would enhance synaptic transmission for cold-conditioned preparations. Furthermore, we postulated that evoked synaptic transmission would be reduced upon acute cold exposure due to the reduced biochemical and molecular events, such as opening of channels and associated protein-protein interactions required for evoked vesicle fusion events. We expected with cold conditioning over time that some biophysical homeostatic regulation would occur to boost evoked synaptic transmission; however, we were not sure that a short time frame of weeks would suffice.

## RESULTS

### Acute exposure to changes in temperature and modulators for low-output NMJs

**Fig. 1.**
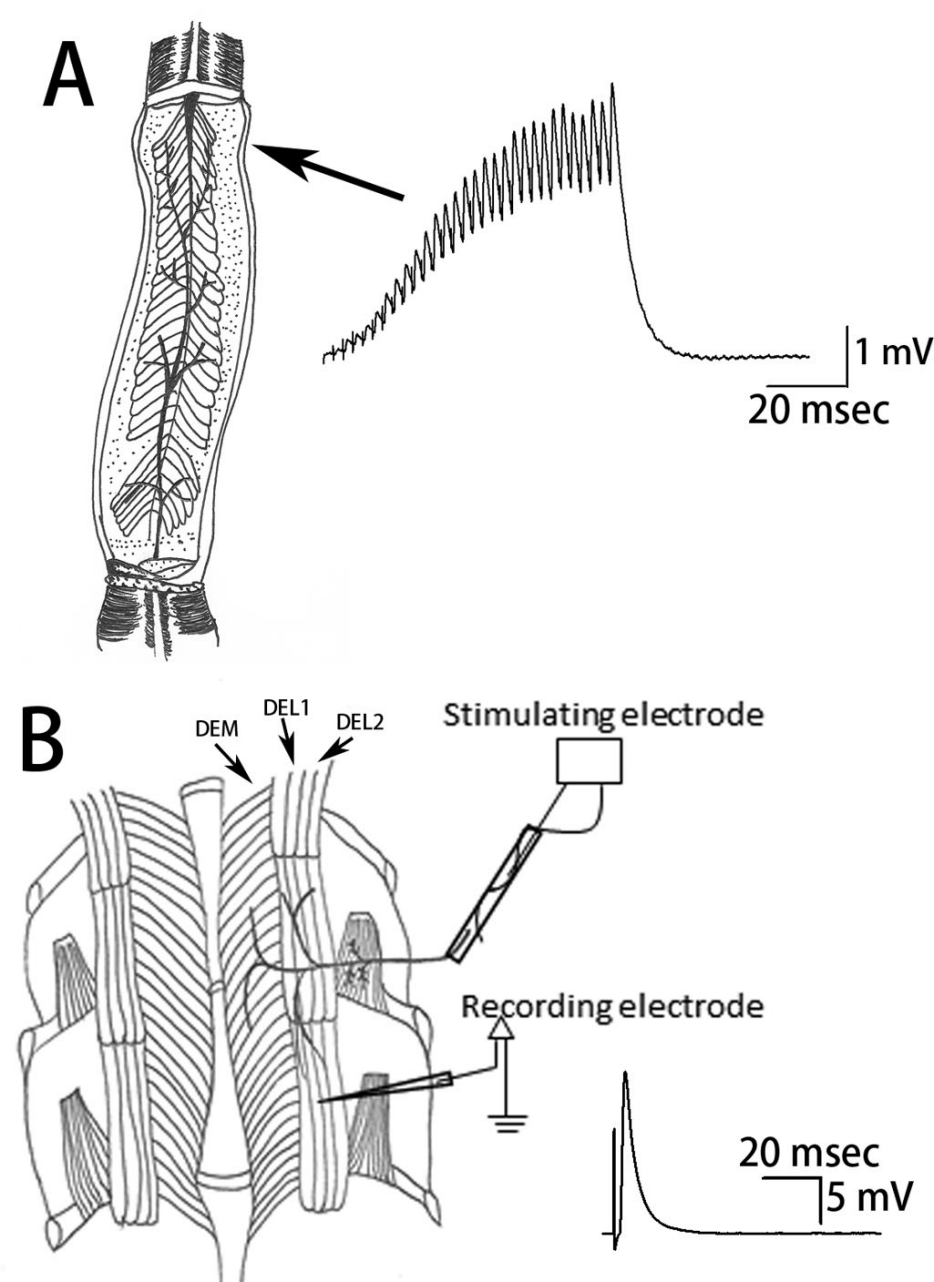
**Walking leg opener and abdomen neuromuscular preparations.** (A) The low output tonic-like neuromuscular junctions on the opener muscle produce an excitatory postsynaptic potential (EPSP) which rapidly facilitates within a 30-pulse train of 40 Hz stimulation. The 10th, 15th, 20th and 30th amplitudes of the EPSP are used to index synaptic efficacy and facilitation. The ventral view looking dorsally at the opener muscle is schematically illustrated. The most distal muscle fibre in each preparation was consistently used to minimise variation among preparations. (B) The high output phasic-like neuromuscular junctions on the deep abdominal lateral extensor muscle (DEL1) produce a large EPSP with a single evoked stimulus. The DEL1 muscle fibre in the segment below the one being stimulated is used since only one motor neuron innervates these fibres from the more anterior segment. The stimulus artefact is recorded, followed by the large amplitude phasic EPSP. The amplitude of the EPSP is used to index the effects of temperature and/or modulation.

**Fig. 2.**
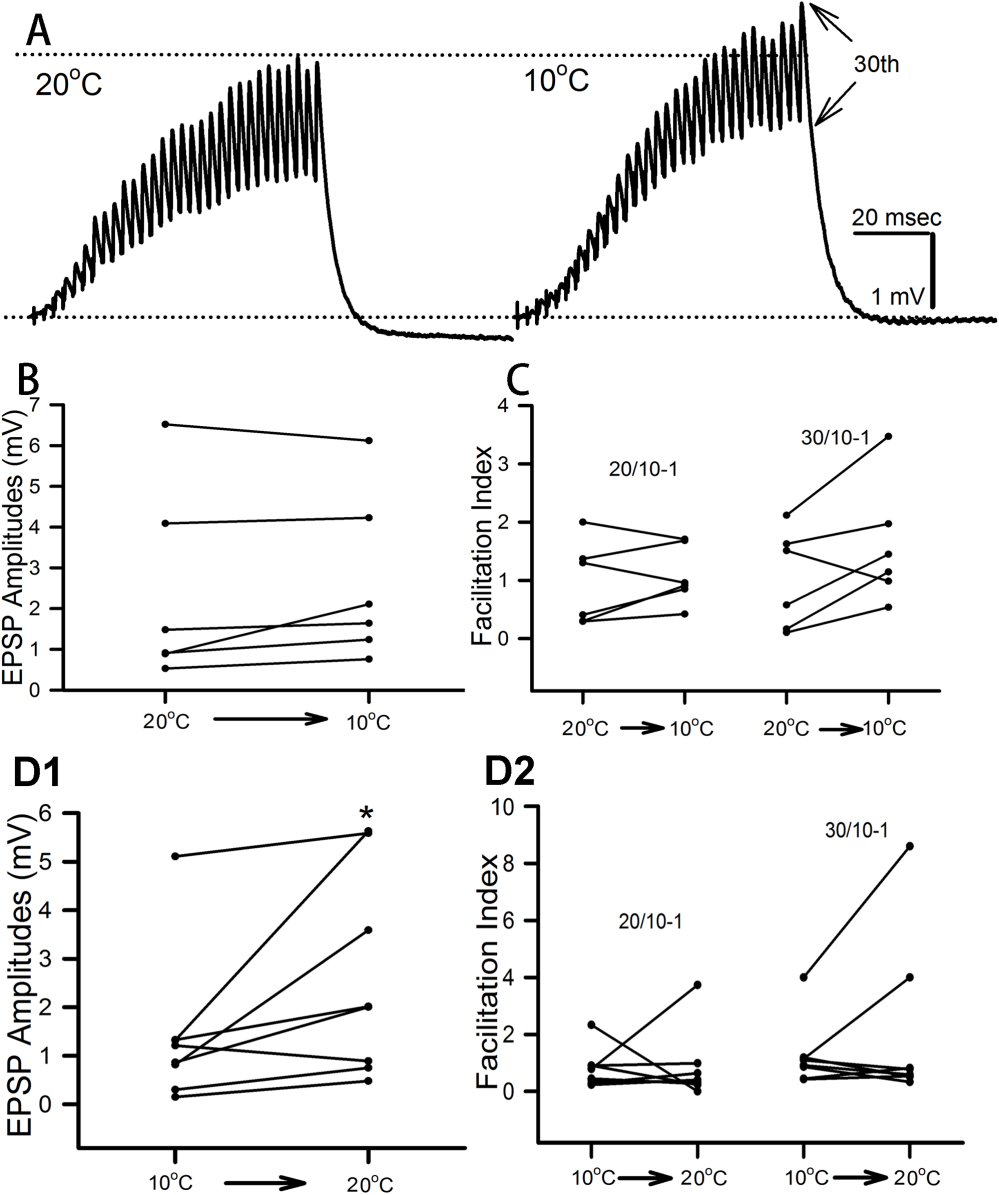
**Effect of acute cold exposure** **on the tonic-like NMJs.** (A) Representative EPSP traces of the changes which occur when exposing a preparation conditioned to 20°C to 10°C. Measurement of the EPSP amplitude is made from the trough preceding the event to the peak of the event, as illustrated for the 30th event within the stimulus train. (B) The amplitude of the 30th EPSP from the 40 Hz stimulation trains before and during acute cold exposure is shown for each preparation. (C) The facilitation index (FI) for each preparation is illustrated. The indices are shown for 20th/10th and 30th/20th EPSPs to indicate the degree in change in facilitation due to the acute cold exposure. The FI is determined by the 30th or 20th EPSP amplitude divided by the 10th EPSP amplitude and subtracting a unit of one to ensure that, if no facilitation occurred, the FI would be zero. The effect of chronic cold conditioning at 10°C on the tonic NMJs when exposed to 20°C on the amplitude of the 30th EPSP from the 40 Hz stimulation trains during the acute cold exposure and after the exposure to 20°C is shown for each preparation (D1). (D2) The facilitation index (FI) for each preparation is illustrated. The indices are shown for 30th/10th and 20th/10th EPSPs to indicate the degree in change in facilitation due to the change from cold conditioning to warm exposure.

The low-output NMJs were assessed on the opener muscle of the first pair of walking legs (Fig. 1A). The effect of rapid exposure (<1 min) to 10°C after acclimating the isolated opener NMJ produced an increase in the 30th EPSP amplitude of the 40 Hz stimulus train in five of the six preparations and a decrease in one preparation (Fig. 2A,B). The facilitation index mimicked the increases in EPSP amplitude with five of the six preparations showing an increase for the 30th/10th index (Fig. 2C). The facilitation index for earlier pulses within the stimulus train were not as consistent in response to acute temperature change. The 20th/10th facilitation index showed mixed results, with four preparations increasing in facilitation and two showing a reduction at the colder temperature (Fig. 2C). This suggests a plateau in the responses was not obtained by the 20th pulse for the responses not demonstrating an increase in the index.

For the crayfish acclimated to 10°C, seven out of eight preparations presented an increase in the 30th EPSP amplitude (Fig. 2D1). This is quite different from the acute change from 20°C to 10°C; the change from 10°C to 20°C resulted in only two of the seven preparations increasing the 30th/10th facilitation index (Fig. 2D2) with mixed results for the facilitation of the earlier EPSPs within the train (20th/10th).

**Fig. 3.**
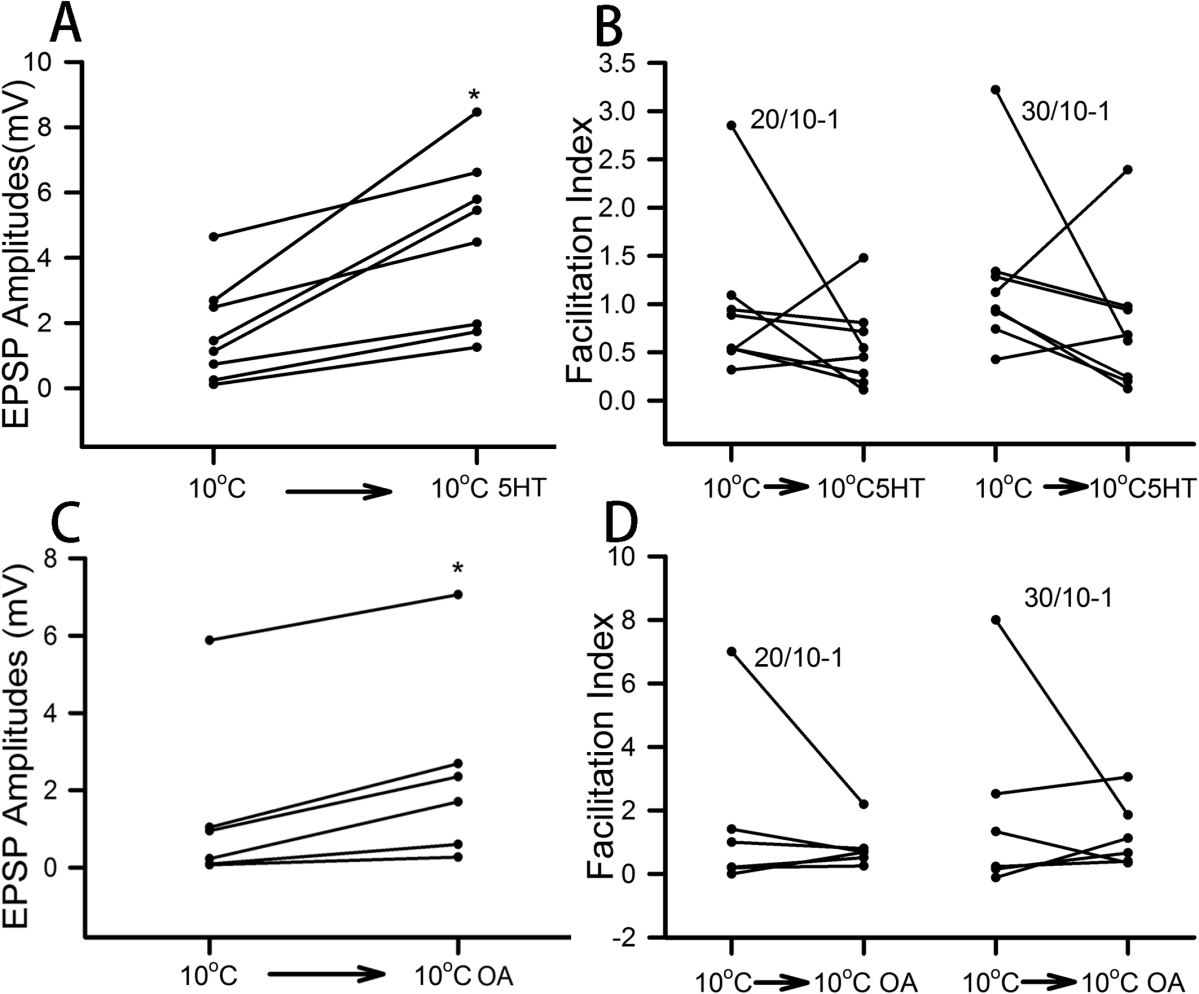
**Effect of serotonin (5-HT) or octopamine (OA) on the synaptic efficacy of chronic cold 10°C-acclimated preparations.** The amplitude of the 30th EPSP from the 40 Hz stimulation trains during chronic cold exposure is shown for each preparation before and during exposure to 5-HT (A) and OA (C). The facilitation index (FI) for each preparation is illustrated before and during exposure to 5-HT (B) and OA (D). The indices are shown for 30th/10th and 20th/10th EPSPs to indicate the degree in change in facilitation due to exposure to the modulators. (*P<0.05, Sign test).

**Fig. 4.**
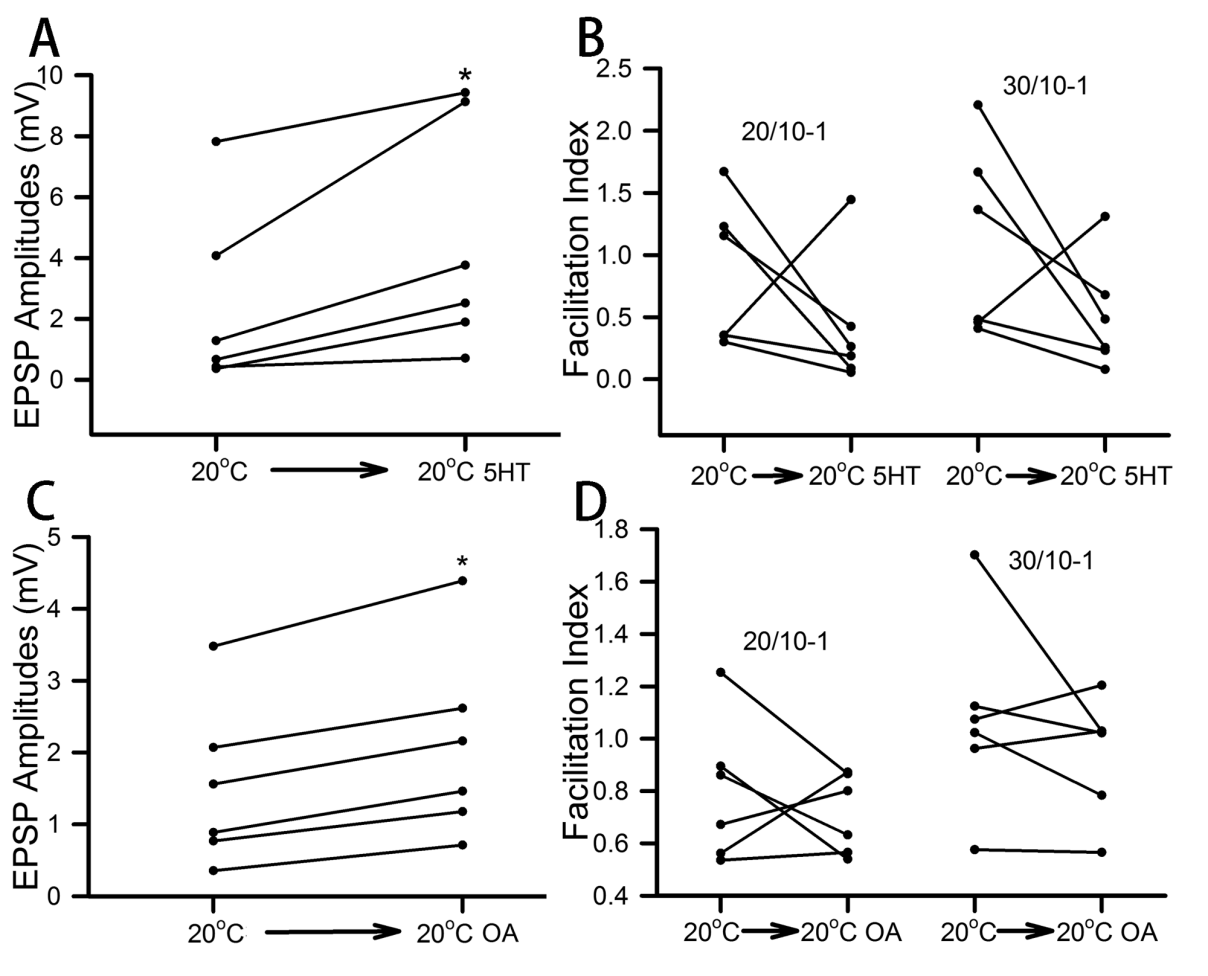
**Effect of serotonin (5-HT) or octopamine (OA) on the synaptic efficacy of 20°C-acclimated tonic NMJs.** The amplitude of the 30th EPSP from the 40 Hz stimulation trains is shown before and during the exposure to 5-HT (A) and OA (C). The facilitation index (FI) for each preparation is illustrated before and during exposure to 5-HT (B) and OA (D). (*P<0.05, Sign test).

The effect of 5-HT (100 nM) and OA (100 nM) on the opener NMJs was consistent in enhancing the amplitudes of the 30th EPSP within the stimulus train for every preparation from the crayfish acclimated to 10°C (Fig. 3A,C; *P*<0.05). However, the facilitation indices resulted in mixed responses for the 30th/10th and 20th/10th for both 5-HT and OA exposure (Fig. 3B,D). Like for the 10°C-acclimated crayfish, the 20°C-acclimated animals all exhibited increases in the amplitude of the 30th EPSP of the stimulus train for both 5-HT (100 nM) and OA (100 nM) (Fig. 4A,C; *P*<0.05). The facilitation index for the 30th/10th and 20th/10th decreased with 5-HT in five of the six preparations (Fig. 4B). The results were not as consistent for exposure to OA in the measurements of the facilitation indices (Fig. 4D).

### Acute exposure to changes in temperature and modulators for high-output NMJs

**Fig. 5.**
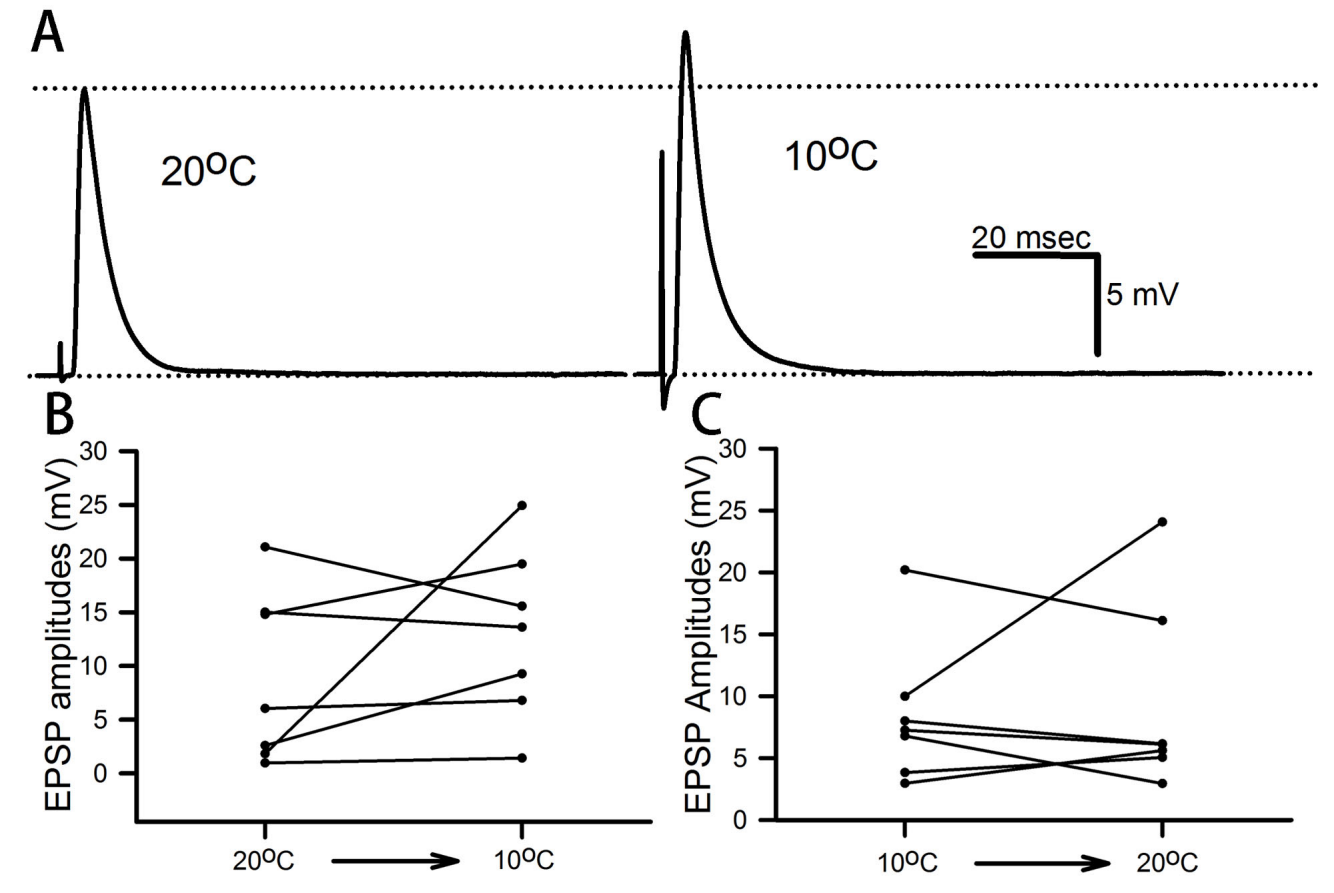
**Effect of acute cold exposure on the phasic NMJs.** (A) Representative EPSP trace of exposure to 10°C for a preparation conditioned to 20°C. (B) The amplitude of the EPSP before and during the acute cold exposure is shown for each preparation. (C) The amplitude of the EPSP before and during acute exposure to 20°C of preparations conditioned to 10°C.

The phasic (high-output) NMJs of the DEL1muscle (Fig. 1B) produced an increase in the EPSP amplitude in four of the six preparations upon acute exposure to 10°C for the 20°C-acclimated crayfish (Fig. 5A,B). The results were varied not only in the initial amplitude of the EPSPs, but also in the degree of change upon exposure. Similarly, mixed results occurred for the preparations of 10°C-acclimated crayfish acutely exposed to 20°C saline (Fig. 5C).

**Fig. 6.**
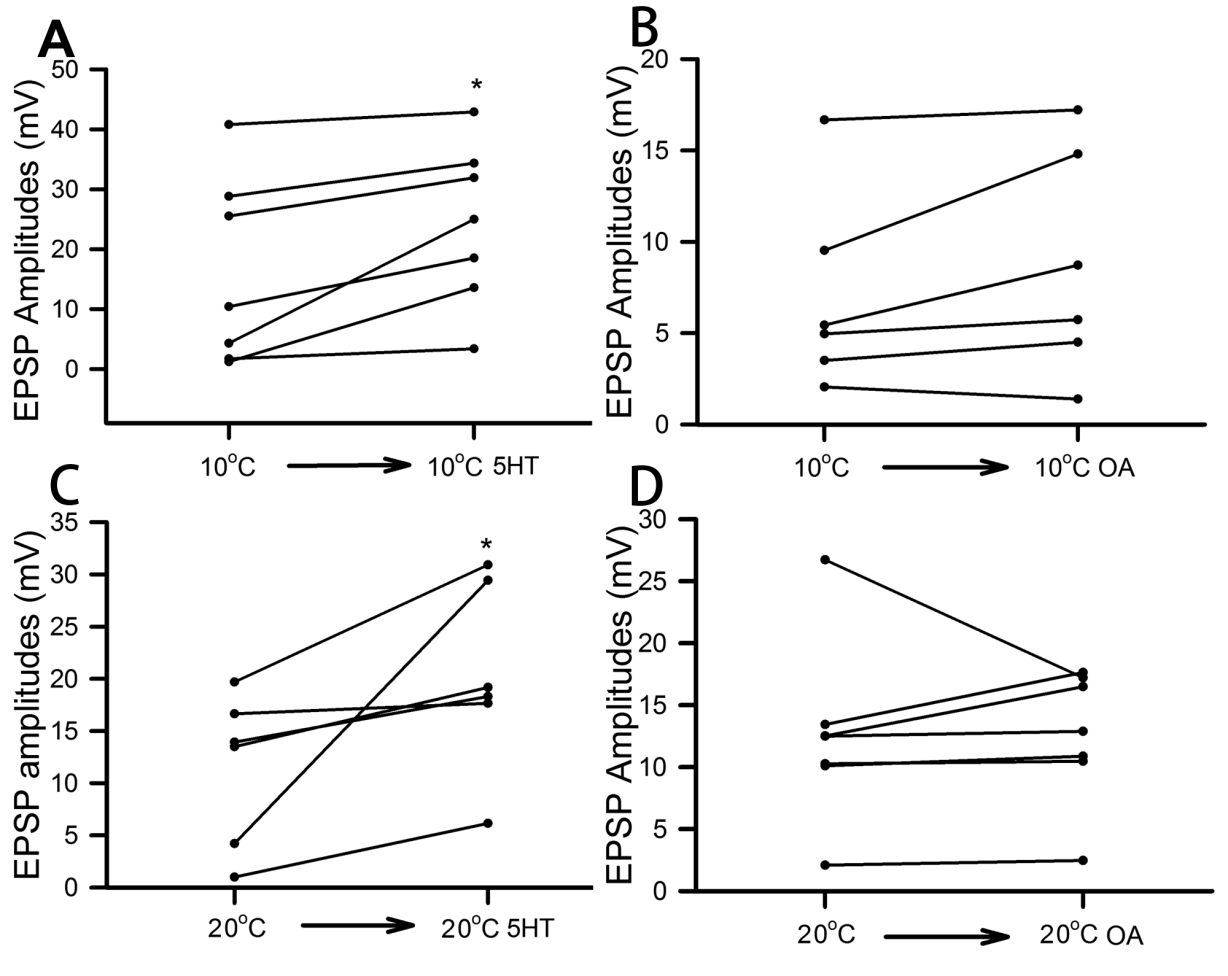
**Comparison of the effect of 5-HT or OA on the EPSP amplitudes for phasic NMJs for crayfish acclimated at 20°C or 10°C temperatures.** Crayfish were acclimated at 10°C and exposed to 5-HT (100 nM) (A) or octopamine (100 nM) (B). Similarly, crayfish were acclimated at 20°C and exposed to 5-HT (100 nM) (C) or octopamine (100 nM) (D). (*P<0.05, Sign test).

As observed for the low-output tonic NMJs, the high-output phasic NMJs all resulted in increases in the amplitude of EPSPs upon exposure to 5-HT (100 nM) for crayfish acclimated at both 10°C and 20°C (*P<0.05; Fig. 6A,C). However, one preparation in each of the 10°C- (one out of six preparations) and 20°C-acclimated (one out of seven preparations) crayfish resulted in a decrease in EPSP amplitude during exposure to OA (100 nM) (Fig. 6B,D).

### Muscle fibre resting membrane potential, input resistance and OA concentration with changes in temperature

The resting membrane potentials obtained from all the tonic and phasic NMJs used in this study were compared at the acclimated temperatures of 10°C and 20°C. Both muscle preparations were significantly depolarised at 10°C relative to 20°C (tonic: *N*=16, 19, unpaired *t*-test, *P*<0.05; phasic: *N*=19, 20, unpaired *t*-test, *P*<0.05).

**Fig. 7.**
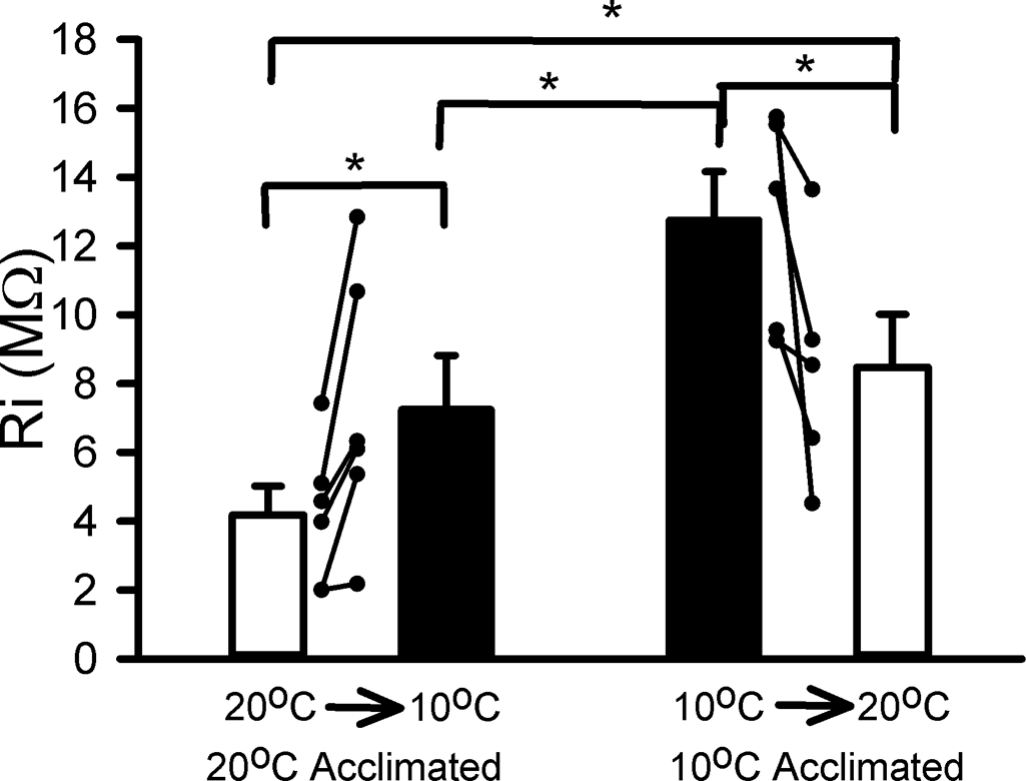
**Muscle fibre input resistance for muscle conditioned to 20°C and exposed to an acute change to 10°C, as compared to a muscle chronically conditioned to 10°C and exposed acutely to 20°C.** Individual preparations are shown, along with an averaged response (±s.e.m.) (*P<0.05, Sign test).

The input resistance of the distal muscle fibres from the walking leg opener muscle preparation was determined upon acute changes in temperature for 10°C- and 20°C-acclimated crayfish (Fig. 7). The 20°C-acclimated crayfish all exhibited an increase in input resistance upon exposure to 10°C (*P*<0.05; signed rank, *N*=6). The 10°C-acclimated crayfish all exhibited a decrease in input resistance upon exposure to saline at 20°C (*P*<0.05; signed rank, *N*=5). The wide range in values is likely due to the different sized muscle fibres among the different preparations; hence the reason for examining changes for given fibres while acutely exposed to a change in temperature. It is interesting to note that even though relatively similar sized crayfish and legs were used for the experiments, the 20°C-conditioned crayfish had a lower input resistance then the acutely exposed 20°C preparations conditioned at 10°C (*P*<0.05; *t*-test).

The resting membrane potential of the opener muscle trended toward depolarisation as the temperature is lowered in the 20°C-acclimated crayfish (*P*=0.063; signed rank test) and the membrane hyperpolarises as the temperature rises in the 10°C-acclimated crayfish (*P*<0.05; signed rank *t*-test) (Table 1). The Q10 values (i.e. the change in values for every 10°C change) for the resting membrane potentials also varied depending on the direction of the temperature change. For the opener muscle starting at 20°C and being exposed to 10°C, the value is 1.37. However, starting at 10°C and going to 20°C, the value is 2.50. The directional shifts are even larger for the DEL1 muscle, with a change from 20°C to 10°C producing a Q10 of 3.26 and 10°C to 20°C producing a Q10 of 1.21.

**Table 1.**
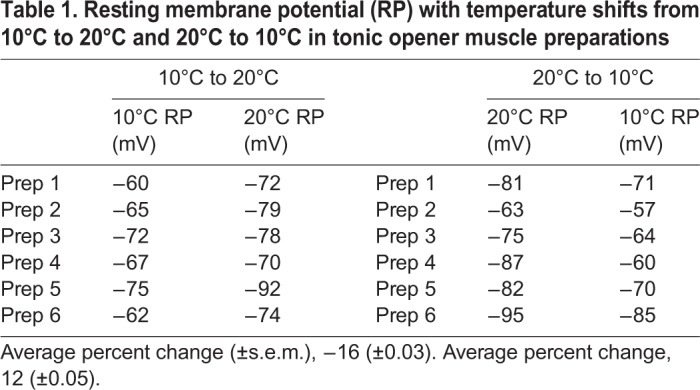
**Resting membrane potential (RP) with temperature shifts from 10°C to 20°C and 20°C to 10°C in tonic opener muscle preparations**

Lastly, analysis of the amount of OA in the haemolymph samples obtained from six crayfish acclimated at either 10°C or 20°C revealed that the 10°C-acclimated crayfish contained a higher level of OA (30.48 ng/ml±9.3; mean±s.e.m.) than the 20°C-acclimated crayfish (7.08±1.6; mean±s.e.m.; *P*<0.05; paired *t*-test). The 5-HT levels were too low in some samples to be reliably measured and compared; thus, they are not reported.

## DISCUSSION

In this study, it was demonstrated that a temperature shift from 10°C to 20°C increases EPSP amplitudes at the NMJs of the opener (tonic) preparation. However, temperature shifts (20°C to 10°C or 10°C to 20°C) did not have a consistent effect on EPSP amplitudes for the abdominal DEL1 phasic NMJ. When tonic and phasic NMJs were exposed to 5-HT, EPSP amplitudes showed a substantial increase in both 10°C- and 20°C-acclimated crayfish. Exposure to OA in the opener preparations also resulted in an increase in EPSP amplitudes in both 10°C- and 20°C-acclimated crayfish. However, exposure to OA resulted in a consistent increase in the abdominal preparations at 10°C, while at 20°C the acclimated crayfish showed varied results. The input resistance of the muscle membrane increased and the resting membrane potential depolarised with a temperature change from 20°C to 10°C. HPLC analysis showed an increase of OA concentration in the haemolymph of 10°C-acclimated crayfish. This increased OA may aid the neuromuscular junction to remain functional at low temperatures. These studies showed a difference in responsiveness to modulators enhancing neuromuscular junction transmission between cold and warmer conditions.

### Behavioural effects to cold

If given a choice, some crustaceans show a behavioural preference for a particular thermal environment (Forward, 1990; Hobbs, 1981; Payette and McGaw, 2003). However, the environment can change rapidly enough that an animal may not have a chance to move to more suitable conditions, causing acute exposure to the new conditions. Thus, survival requires the ability to change physiologically in one aspect to permit sufficient behavioural control to forage and avoid predation. Crustaceans unable to undergo diapause (Hand et al., 2016) but requiring a functional nervous and muscular system for survival, would need the ability to maintain cellular responses. The adaptive physiological processes are unique depending on the crustacean species. *Macrobrachium rosenbergii*, commonly referred to as the Malaysian prawn, die when rapidly or slowly exposed to 10°C (Chung et al., 2012) because cardiac and neural function ceases at this temperature. In contrast, *P. clarkii* can survive and respond to sensory stimuli. The small freshwater crustaceans *Niphargus rhenorhodanensis* and *Niphargus virei* (both cave crustaceans) and a close relative of these two cave-based species, *Gammarus fossarum* (surface crustacean), are known to survive to −2°C (Issartel et al., 2005). The estuarine crab (*Hemigrapsus crenulatus*) is found in nature within cold environments at around 2.5°C (Cumillaf et al., 2016). Even though various crustaceans are known to live in cold environments and have biochemical differences which likely account for their survival, the physiological reactions to acute changes in temperature have not been investigated. A limited ability to survive changes in temperature may be a reason for the local endemic nature of some species as compared to hardy invasive species' ability to survive a cold environment as well as rapid changes in temperature as simulated in laboratory conditions (Veselý et al., 2015). However, animals may choose ecological niches, such as burrows, which do not expose them to harsh environmental conditions to survive (Jakobs et al., 2015).

### Cold-induced cellular responses

There are various aspects to consider in conditioning to cold, such as cold hardening, when comparing with the effects of an acute change within a few minutes of cold exposure. The effects on changes in osmolality of the haemolymph, gene expression, transcriptome differences, amino acid composition and concentrations of sugars for cold hardening and longer-term cold exposures have been intensively investigated in insects (Colinet et al., 2010; Czajka and Lee, 1990; Overgaard et al., 2007; Teets et al., 2012; MacMillan et al., 2016) and bacteria (Phadtare and Severinov, 2010). In rapid exposure to cold, the cell membrane does not have time to alter its composition; thus, ion channels, pumps and receptors need to function in a potentially more rigid environment when going from 20°C to 10°C. The physiological function of the proteins may be quite different when the membrane has time to undergo changes in composition and cold shock proteins have had time to be expressed. The ability of animals to survive gradual exposure to cold compared to a rapid change in temperature is well known in insects, but little attention has been given to the physiological differences in the synaptic transmission of animals in rapid (<5 min), as compared to hours or days of, cold exposure.

In an intact animal, initiating a movement involves a neural command and activation of descending neural circuits to then activate motor neurons. The synaptic connections and electric flow along axons, as well as muscle contraction, all involve moving proteins as ion channels, ion binding proteins, vesicular docking-fusion complexes, receptors, pumps, exchangers and proteins associated with muscle contraction and relaxation. The effect of temperature on neural circuits has been investigated in the crustacean stomatogastric nervous system for preparations acclimated to different temperatures (Marder et al., 2015; Städele et al., 2015). The function of NMJs and the development of muscle tension in crustacean preparations (crabs and crayfish) are also known to be influenced differently if the intact animal is acclimated to differing temperatures. Muscle fibres in the claws of stone crabs and blue crabs show cold acclimation and have a higher input resistance at 8°C than non-cold acclimated crabs. This higher input resistance results in a broadening of EPSPs which enhances EPSP summation, muscle fibre depolarisation and muscle force (Fischer and Florey, 1981; Hamilton et al., 2007). The wide range in the amplitudes of the EPSPs among the preparations measured in the study herein for a given condition can be for various reasons. Holding crayfish in a laboratory setting can result in altered EPSP amplitudes and rates of depression in the responses (Bradacs et al., 1997). This might be due to the reduced amount of movement by the animal. Seasonal changes are also noted in the literature to produce differences in EPSP amplitudes (Lnenicka and Zhao, 1991). However, such differences were controlled in this study as the crayfish were housed over the same period and season. The amplitudes of the EPSPs can also vary among preparations due to muscle fibre dimensions, which can affect input resistance of the fibre (Atwood, 1982a,b; Atwood and Parnas, 1968; Baierlein et al., 2011; Cooper et al., 1995). In addition, these are wild caught crayfish with unknown life history and health status. Given they are housed individually and held in the laboratory setting as isolates for at least 2 weeks prior to experimentation and for the duration of the cold conditioning, the independent variables are controlled for what is manageable in order to address the effects of temperature altering the EPSP amplitudes.

The muscle plasma membrane and ion channels on the muscle membrane also contribute to the synaptic strength. The ability of the plasma membrane to make changes in its composition likely accounts for the differences in membrane resistance for acute changes in temperature for non-acclimated and acclimated animals (Chapelle, 1977, 1978; Chapelle et al., 1979). The increase in input resistance for 20°C-acclimated crayfish exposed to acute saline change at 10°C would suggest that leak channels do not allow ions to pass as readily. In comparison, 10°C-conditioned animals showed a decrease in input resistance during acute exposure to 20°C. The increased input resistance at 10°C would in part account for some of the increase in the amplitude of EPSPs at 10°C. The 10°C-acclimated animals had a slightly lower input resistance on average, which may account for changes in the composition of the membrane. The potential changes which can occur are likely similar to those well documented for insects as reviewed in Teets and Denlinger (2013).

In a cold environment, the ability of the proteins to function could be decreased due to improper or ridged folding. Therefore, many aspects of synaptic transmission on both presynaptic and postsynaptic sides could be affected by temperature changes as there are hundreds of proteins involved (Dieterich and Kreutz, 2016; Storey and Storey, 1988). One of the most critical issues is the disruption to the electrochemical gradients of ions, which leads to loss of neural activity and neuromuscular coordination (Friedlander et al., 1976; Hayward et al., 2014). In our study, acute and chronic cold exposure caused depolarised resting membrane potentials. This depolarised resting membrane potential could be explained by several factors. The Q10 for the Na^+^/K^+^ pump Q10 is approximately 2.4 in sea urchins (Leong and Manahan, 1997). Furthermore, several papers (Andersen et al., 2013; MacMillan et al., 2015; MacMillan and Sinclair, 2011) indicate that in insect cold exposure, potassium will leak towards the haemolymph causing a progressive increase in extracellular potassium. The reduction in Na^+^/K^+^ pump activity and increased extracellular potassium concentrations can lead to depolarisation of the membrane potential. This may explain the depolarisation that we observed in the acute exposure to 10°C. Moreover, with crustaceans acclimated to different temperatures, there are changes in potassium ion concentrations in the haemolymph at different temperatures. Legarra et al., (1984) found that potassium concentrations decreased with lower temperatures in *P. clarkii* crayfish. Wong and Freeman (1975) also showed that the potassium concentration in the haemolymph is decreased with lower temperatures. Combining the effect of lowered extracellular potassium concentrations with decreased Na^+^/K^+^ pump function at lower temperatures, it is feasible that the resting membrane potential was depolarised with chronic cold exposure in our study. It may indicate that decreased Na^+^/K^+^ is playing a bigger role than the possibly lowered potassium concentrations in the chronic cold-exposed crayfish.

### Alterations in synaptic responses with temperature

Studies in differing species of crayfish (*Astacus leptodactylus*, *P. clarkii)* and crab (*Ocypode ceratophthalma*, *Carcinus maenas*, *Cancer pagurus*) all show a reduction (i.e. more hyperpolarised) in membrane potential with acutely increasing temperature. The relative change is approximately 1 mV to 1.3 mV/1°C change (Florey and Hoyle, 1976; Harri and Florey, 1976, 1979; Hyde et al., 2015). We observed a similar hyperpolarisation of the muscle membrane potential when changing the temperature from 20°C to 10°C. The reason for this change has not been explicitly addressed in prior reports, but it is likely related to the more negative equilibrium potential of K^+^ (E_K_), since the membrane is more permeable to K^+^ and the resting potential is driven mostly by the E_K_.

With a more negative E_K_, one would also assume a larger EPSP would result in the muscle due to a larger driving gradient for E_Ca_ and E_Na_ for glutamate receptor-induced depolarisation of the crustacean muscle. Crayfish skeletal muscle uses voltage-gated Ca^2+^ channels in the plasma membrane, which contribute to the EPSP and muscle contraction. However, in crabs and crayfish it has been shown that the amplitude of EPSP decreases with increasing temperature past the conditioned temperature of the animal (Florey and Hoyle, 1976; Harri and Florey, 1976, 1979; Hyde et al., 2015). The amplitudes of the EPSP are varied and have been shown to be generally optimal at the temperature to which the animal is conditioned. This maximum amplitude in the EPSP for an acclimated temperature is more commonly apparent for the tonic NMJs than for phasic NMJs (Harri and Florey, 1979). However, in our studies, the 20°C-conditioned animals showed a slight enhancement in EPSP amplitude with acute exposure to 10°C, whereas the 10°C conditioned animals showed an increase with acute exposure to 20°C. The fact that cooling increases input resistance and depolarises the resting membrane potential is well documented in crustacean muscles for crabs and crayfish (Blundon, 1989; Fischer and Florey, 1981; Harri and Florey, 1976, 1979). Cold temperatures are known to result in slower calcium ion movement, as shown in squid axons (Charlton and Atwood, 1979); however, this has not been directly assessed in crayfish nerve terminals. Could slower movement of Ca^2+^ ions impact residual Ca^2+^ levels with repetitive stimulation?

No mechanistic explanations have yet accounted for the differences observed in the maximal EPSP response at tonic and phasic NMJs in acclimated animals. One reason may be that the tonic NMJs, which are more metabolically active, undergo rapid change to homeostatically regulate synaptic efficacy. Perhaps if the acclimation periods in the experimental studies were extended, the phasic NMJs may also show a shift in optimal amplitude of EPSP to the conditioned temperature. However, there might just be intrinsic differences which do not allow phasic NMJs to shift the EPSP amplitude. In natural settings, crayfish show seasonal differences in phasic EPSP amplitudes. In the winter, the phasic NMJs produce larger EPSPs which are depression prone, compared to in the summer when the crayfish are more active and have smaller amplitude EPSP for phasic NMJs that are also fatigue resistant (Lnenicka, 1993; Lnenicka and Zhao, 1991). Activity-dependent changes have also been induced in laboratory conditions with electrical conditioning of the phasic motor neurons (Cooper et al., 1998). Thus, the phasic NMJs are dynamic enough to change their synaptic output with evoked activity but adjusting to the acclimated temperature for a maximal response must involve different processes. The seasonal effects are apparently species-dependent, as the crab *C.* *pagurus* does not show seasonal effects, in contrast to *C.* *maenas*, in regard to muscle physiology (Hyde et al., 2015), but both crabs *Menippe mercenaria* (Say) and *Callinectes sapidus* (Rathbun) show changes with seasonal temperature changes, suggesting different adaptive advantages (Blundon, 1989). However, it is difficult to separate the direct effect of temperature from activity-dependent change, as the behaviour of the animal changes with environmental temperature. The types of changes which occur in synaptic transmission with cold exposure in crustaceans has recently been reviewed (Zhu and Cooper, 2018).

### Hormonal effects with changes in temperature

Considering how rapidly hormones can act on altering synaptic transmission and muscle tension, it is realistic to entertain the possibility that rapid cold stress exposure may result in altered release of biogenic amines or peptides to potentially compensate for altered synaptic function. We are not aware of any reports in which biogenic amines or peptides have been measured in the haemolymph of insects or crustaceans during exposure to cold, except for Zhu et al. (2016). Zhu et al. showed that OA decreased in cold-acclimated (10°C) larval *Drosophila* relative to acclimation to 20°C. We are also not aware of any reports on changes in biogenic amine levels in cold conditions relative to warmer temperatures for a crustacean. The increase in OA for the cold-conditioned crayfish may enhance the skeletal muscle to produce and increase the force of contraction, since it is known to do so in insects and crustaceans in warmer temperatures (Fischer and Florey, 1987). In addition, the increase in EPSP amplitude upon exposure to OA for the 10°C-acclimated crayfish indicates that the preparations could respond to an even greater concentration in the intact system. It is unlikely that the effect of OA in the conditioned crayfish would have dissipated within five minutes of preparing the preparation for electrophysiological recording and exposure to 100 nM of exogenously applied OA for a test in responsiveness. The larger increases in EPSP amplitude in the isolated preparations for 20°C acclimated crayfish may be indicative of the lower concentration in the intact animal, thus resulting in an increased sensitivity when exposed. In previous studies, 5-HT and OA displayed a strong ability to enhance presynaptic and postsynaptic processes at the neuromuscular junction in crustaceans. Octopamine is able to increase contractile force in the crustacean skeletal muscle (Fischer and Florey, 1987) as well as alter muscle force and synaptic structure in insects (Koon and Budnik, 2012). It was shown for the isolated lobster pyloric muscle that a decrease in its rhythmicity occurred at warmer temperatures; however, in the presence of dopamine, the activity was maintained (Thuma et al., 2013). The authors suggested that within the animal dopamine may be responsible for the animal maintaining similar consumption of food in warm and cold conditions. Thus, in this case, an increase in a modulator is helping the NMJs and animal to function better in a warm environment.

For the 10°C-acclimated crayfish, we noticed that with 40 Hz stimulating pulses to the opener motor nerve, with a facilitating train, the 30th EPSP was almost undetectable when bathed in 10°C saline. However, upon adding 5-HT or OA, the amplitude of the EPSP increased. Initially, it appeared as if the nerve was not being stimulated since the EPSPs were so small in saline alone. However, before removing the leg, the animal was able to close the claw in the walking leg robustly when disturbed. It would be interesting to test the force of contraction of the claw in the first pair of walking legs in intact animals for the two acclimated temperatures to see if force generation is different. At 20°C, the same stimulus train always revealed a large amplitude EPSP by the 30th pulse within the train. Despite the lower EPSP amplitude in cold conditions, the force of muscle generation may be similar since the OA level is increased in the 10°C-acclimated crayfish. As shown in larval *Drosophila*, the shape of the evoked EPSP is not affected by OA despite an increase in force generation in the muscle. Subsequent studies in cold-acclimated crayfish and effects on muscle tension development with OA exposure would potentially answer this question. However, upon acclimating the animals to cold, the animal will raise the endogenous OA levels. A pharmacological means of blocking OA synthesis in 20°C- and 10°C-acclimated crayfish and then examining the isolated leg's ability to generate force when exposed might be feasible, assuming that receptor expression level would not be differentially regulated. In addition, the rate of exposure to modulators, either by exogenous application or endogenous release, can have varied consequences (Teshiba et al., 2001).

When changing the stimulation pulse from 40 Hz to 60 Hz, the EPSP amplitudes increase, even if barely detectable at 10°C. This may suggest that lower temperature disrupts the calcium influx in the presynaptic terminal. Therefore, with longer trains of stimulation, more calcium could accumulate in the presynaptic terminals to result in more vesicle fusion events (Cooper et al., 1995; Desai-Shah et al., 2008). We had assumed that the nerve may have failed in being evoked at colder temperature, as occurs in squid axons (Prosser and Nelson, 1981; Weight and Erulkar, 1976), but upon a higher stimulation frequency or with the addition of 5-HT or OA, the EPSPs would increase to measurable levels. Thus, we knew that the axon was indeed being recruited. It was noted in an earlier study (Jacobs and Atwood, 1981) using crayfish in which differences in long-term facilitation was being investigated for cold-exposed animals, as well as isolated NMJ preparations, that synaptic responses are more pronounced within the animal.

### Areas for further development

Gaining a better understanding of the general effects on synaptic physiology of acute and longer-term cold exposure will potentially aid in applications of therapeutic hypothermia in humans (Sadaka et al., 2013; Shankaran, 2009; Yenari and Han, 2012). In hibernating animals, which lower their body temperature, a cold-induced protein shows promise in protecting neurons from damage and aids in synapse reformation during the rewarming of hibernating mammals (Peretti et al., 2015). It is known that synapses can fade away with cold and can reappear not only in hibernating mammals (Peretti et al., 2015), but also in arthropods (Brandstatter and Meinertzhagen, 1995).

In the temperature shift from 10°C to 20°C, we observed a significant increase in the EPSP amplitude, which may be expected with a larger driving gradient from a more negative resting membrane potential; however, one would not expect an increased EPSP amplitude with a reduced input resistance. This indicates that these biophysical components involved in synaptic transmission might be justified for 20°C, as it is more closely aligned to the optimal temperature range of this species of crayfish. Perhaps now, adding the potential effects of a modulator such as OA and drawing from findings in other invertebrates that muscle force is increased with OA (Evans et al., 1975; Ormerod et al., 2013) and synaptic responses are increased (Djokaj et al., 2001; Fischer and Florey, 1987), these effects could account for *in vivo* abilities of the NMJs to remain active in a cold environment.

## MATERIALS AND METHODS

### Husbandry

These experiments were all performed using *P. clarkii* crayfish measuring 6–8 cm in body length (obtained from Atchafalaya Biological Supply Co., Raceland, USA). Each animal was individually stored in an aquatic facility and was fed commercial fish food pellets (Aquadine) – marketed as ‘shrimp and plankton sticks: sinking mini sticks’– for at least 2 weeks prior to experimentation. All animals were fed equal portions at equal time intervals. The experimental paradigm was designed to have two conditions: one where crayfish were maintained at room temperature (20–21°C) and another where a group of crayfish underwent cold conditioning by being exposed to 15°C for 1 week and then 1 week at 10°C prior to experimentation.

### Dissection

Both preparations, the opener and the DEL1, have been investigated in relation to the muscle phenotype (i.e. phasic/fast and tonic/slow) and innervation characteristics. Use of the opener muscle for studying synaptic transmission has been previously reviewed (Cooper and Cooper, 2009). The use of the most distal muscle fibres in the preparation provides a reference for consistency among preparations (Cooper et al., 1995). The tonic phenotype of the innervation of these fibres is low output and fatigue resistant but has pronounced facilitation (Crider and Cooper, 1999, 2000). The phasic phenotype of the innervation of the DEL1 muscle is of high output and it is fatigue prone. The DEL1 muscle is used by the animal to extend the abdomen. The NMJs in these two muscles types show enhanced synaptic plasticity and altered responsiveness to biogenic amines (Cooper et al., 1998; Griffis et al., 2000).

The NMJs in the opener muscle that were examined were the excitatory innervation of the most distal muscle fibres on the opener muscle of the first and second walking legs (Mykles et al., 2002). For the phasic NMJ, we made use of the innervation of the deep lateral extensor muscle (DEL1) in the segment adjoining the segmental root being stimulated (Cooper et al., 1998; Nguyen and Atwood, 1990). The dissection to expose and selectively stimulate the excitatory motor neuron of the opener muscle is described in textual and video format (Cooper and Cooper, 2009). In brief, the excitatory neuron is isolated from the inhibitor neuron and stimulated in the meropodite segment. The most distal fibres of the opener were always used for recording synaptic responses. The stimulation paradigm consisted of providing a train of 30 pulses at 40 Hz with 10 s between trains. An average of 10 to 20 trains in each of the conditions was used for measures of EPSPs (Fig. 1A). The rationale for using 30 pulses in a train is that by the 30th pulse a plateau in the amplitude of the EPSP potentials is usually reached at room temperature for the opener NMJs (Wu and Cooper, 2012).

The dissection procedure for exposing the phasic abdominal muscle (DEL1) is also described in text and video format (Baierlein et al., 2011; Robinson et al., 2011). The animals were euthanised in less than 5 s by rapid decapitation followed by removal of the abdomen. The intracellular muscle recordings were taken from the most medial DEL1 muscle fibres in the abdominal segment most distal to the segmental nerve being stimulated. These fibres are innervated in part by a descending branch of a single motor neuron that also innervates the DEL1 muscle of the segment in which the segmental nerve was stimulated (Lnenicka and Atwood, 1989; Mercier and Atwood, 1989; Parnas and Atwood, 1966; Sohn et al., 2000). Using the more distal segmental DEL1 muscle allowed the recordings to be made from a single identified motor neuron without the possibility of other phasic motor neurons compounding the excitatory responses, as the DEL1 and DEL2 muscles within a segment are innervated by multiple excitatory phasic motor neurons (Fig. 1B).

### Electrophysiology

Dissected preparations were maintained in crayfish saline, a modified van Harreveld's solution (in mM: 205 NaCl, 5.3 KCl, 13.5 CaCl_2_·2H_2_O, 2.45 MgCl_2_·6H_2_O, and 5 HEPES adjusted to pH 7.4). All intracellular recordings were made with a sharp glass electrode filled with 3.0 M KCl, and both recorded and analysed via Scope and LabChart software (ADInstruments).

The amplitudes of the EPSPs elicited by the motor nerve were monitored. Intracellular responses were recorded with a 1× LU head stage and an Axoclamp 2A amplifier (Molecular Devices, Sunnyvale, USA). EPSP amplitudes for the opener muscle were measured from the start of the rise to the peak of the given event (i.e. the trough between the 29th and 30th EPSP events to the peak of the 30th EPSP event). The amplitudes for the 10th, 20th and 30th EPSP events were used for calculating the facilitation index (FI). In order not to fatigue the NMJ of the DEL1 muscle, the segmental nerve was stimulated at 0.5 Hz for 10 to 20 events in various conditions so that an average amplitude of the EPSPs could be measured. The appropriate motor nerves were stimulated with a suction electrode via an S88 Stimulator (Astro-Med, Inc., USA).

Membrane resistance in muscle cells was obtained by delivering pulses of current and measuring the voltage changes in a plateau of the response. This was performed by impaling each sampled fibre in its central region with a microelectrode and passing current pulses of 500 ms duration within the range of −5 to +5 nA (±0.5 nA increments altering + and − steps). The slope of the voltage-current plot was used to obtain the input resistance as previously described (Cooper et al., 1998). The membrane resistance was determined from the slope of the best fit linear curve.

For the room temperature experiments, recordings were made in a standard laboratory with saline maintained at 20°C in a water bath. The recording dish used for changing saline to 10°C held 30 ml of saline. The saline to be added was maintained in a beaker placed in an ice bath while the temperature was monitored with a digital thermal probe placed in the middle of the saline. Saline kept at 20°C was added to the beaker to maintain a temperature of 10°C just prior to adding to the recording dish. The relatively large volume of saline in a deep dish resulted in a steady temperature for the rapid measurement of EPSPs and in other preparations to rapidly inject current for measuring membrane resistance. The EPSP measurements took less than 30 s since the recording setup was in place for the warmer temperature condition. Going through positive/negative current injections only required about a minute on the storage oscilloscope with digital cursors. For the cold conditioned experiments, electrophysiological recordings were made in a walk-in incubator where the crayfish were conditioned at 10°C. The animals were dissected in the cold room with saline maintained in the cold room. For the cold to warm acute exposure, saline kept at 20°C was maintained outside the walk-in environmental chamber and was added quickly to the preparation in the cold room. Likewise, the relatively large volume of saline in a deep dish resulted in a steady temperature for the rapid measurement of EPSPs and in other preparations to rapidly inject current for measuring the membrane resistance. The EPSP measurement took less than 30 s for the recording since the recording setup was in place for the colder temperature condition. Going through positive/negative current injections only required about a minute on the storage oscilloscope with digital cursors.

5-HT and OA were made into a stock of 1 mM aliquots with crayfish saline. On the day of experimentation, the stock aliquot was diluted to 100 nM in crayfish saline and maintained at the temperature required. The saline bath was rapidly exchanged with the modulator by removing the saline with a large volume pipette and replaced rapidly with saline containing the modulator. All chemicals, salts and modulators were obtained from Sigma-Aldrich.

### High-pressure liquid chromatography with electrochemical detection (HPLC-EC) of haemolymph samples

To evaluate the 5-HT and OA levels in the haemolymph, approximately 1 ml haemolymph was drawn. The haemolymph was obtained directly from the haemocoel with an 18-gauge needle in a ventral puncture close to the ventral nerve cord. Haemolymph samples were taken from animals exposed to their respective environment. The haemolymph was mixed 1:1 in the tube containing the HPLC mobile phase and immediately frozen and stored at −80°C until the HPLC could be performed. The quantification of 5-HT and OA levels in the haemolymph was accomplished through HPLC-EC. The samples were analysed at the Center for Microelectrode Technology (CenMeT) and Parkinson's Disease Translational Center of Excellence, University of Kentucky Medical Center, Lexington, Kentucky, USA (40536-0298).

### Statistical analysis

All data are expressed as means (±s.e.m.). SigmaPlot (version 13.0) was employed for statistical analysis. The paired *t*-test was used in some experiments to compare the percentage change before and after altering the temperature and the effect of cold conditioning. As specified in the results section, an unpaired *t*-test was used to compare between treatments in cases where there were two groups that were not paired. In some cases, a non-parametric analysis (Wilcoxon signed-rank) in the direction of change within each individual was used to compare EPSP amplitude differences based on treatments. *P*<0.05 was considered statistically significant and is indicated with an asterisk in the figures.
